# 2-[(2-Methyl­phen­yl)amino]­quinoline-3-carb­oxy­lic acid

**DOI:** 10.1107/S2414314626005547

**Published:** 2026-05-29

**Authors:** Shuyuan Guo, Sihui Long

**Affiliations:** a School of Chemical Engineering and Pharmacy Wuhan Institute of Technology,Wuhan Hubei 430205 People’s Republic of China; University of Aberdeen, United Kingdom

**Keywords:** hydrogen bond, acid–acid dimer synthon, single crystal

## Abstract

In the title compound, C_17_H_14_N_2_O_2_, the dihedral angle between the quinoline ring system and the pendant phenyl ring is 6.19 (6)° and an intra­molecular N—H⋯O hydrogen bond supports the near-planar conformation. In the extended structure, the mol­ecules associate to form centrosymmetric carb­oxy­lic acid dimers linked by pairs of O—H⋯O hydrogen bonds.

## Abstract 2

In the title compound,the dihedral angle between the quinoline ring system and the pendant phenyl ring is 6.19 (6)° and an intra­molecular N—H⋯O hydrogen bond supports the near-planar conformation. In the extended structure, the mol­ecules associate to form centrosymmetric carb­oxy­lic acid dimers linked by pairs of O—H⋯O hydrogen bonds.

## Graphical abstract

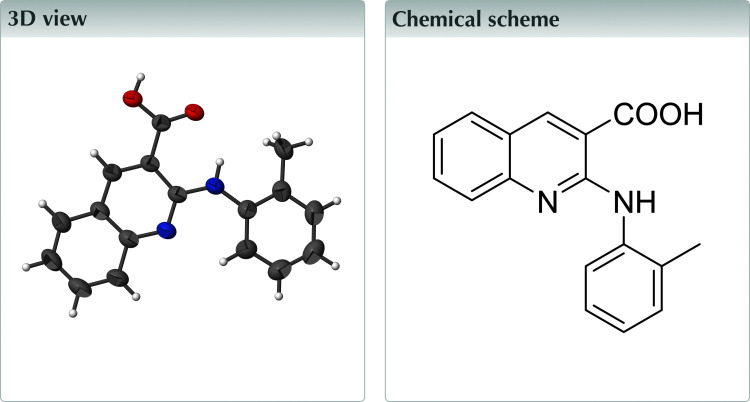

## Structure description

Nonsteroidal anti-inflammatory drugs (NSAIDs) are a class of drugs that do not contain a steroidal structure but possess anti-inflammatory, analgesic, and anti­pyretic effects (Ho *et al.*, 2018 ▸). NSAIDs mainly exert their anti-inflammatory and analgesic effects by inhibiting cyclo­oxygenase (COX), thereby reducing the production of prostaglandins. Salicylic acid was the first NSAID to be discovered (Jiang *et al.*, 2018 ▸). Following salicylic acid, benzoic acid and nicotinic acid derivatives became important NSAIDs, exemplified by tolfenamic acid and clonixin. These anti-inflammatory drugs have been found to exhibit polymorphism. For example, tolfenamic acid has been found to have nine crystal forms (Subaiea *et al.*, 2011 ▸). As part of our studies in this area, we now describe the synthesis and structure of the title compound, C_17_H_14_N_2_O_2_ (**I**).

**Figure 1:**
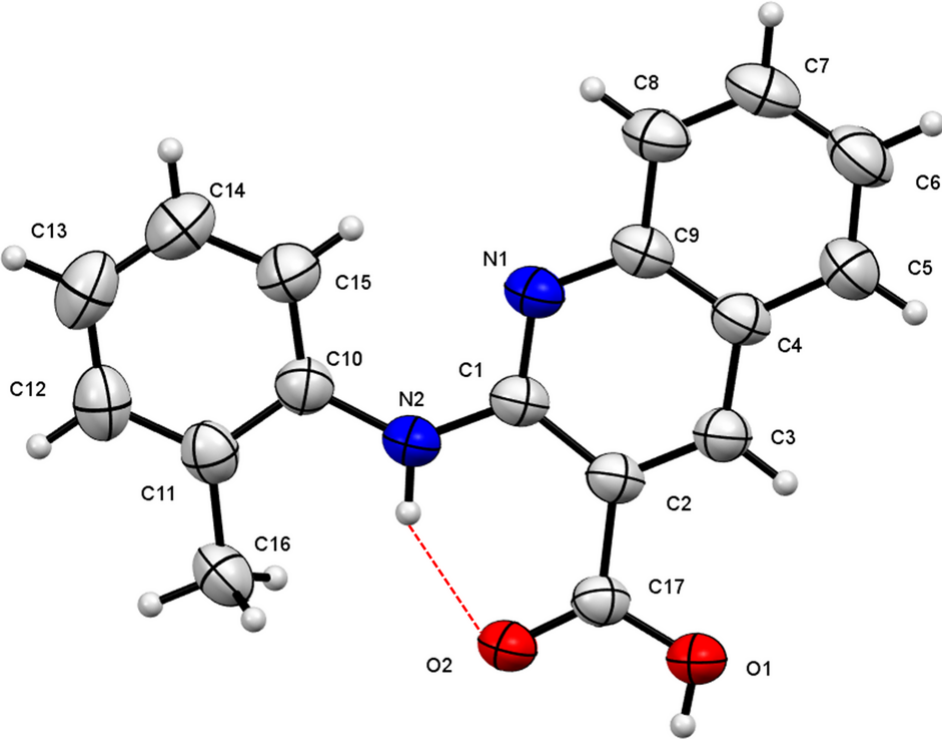
The mol­ecular structure of (**I**) drawn at the 50% probability level. The intra­molecular hydrogen bond is shown as a dashed line.

**Figure 2:**
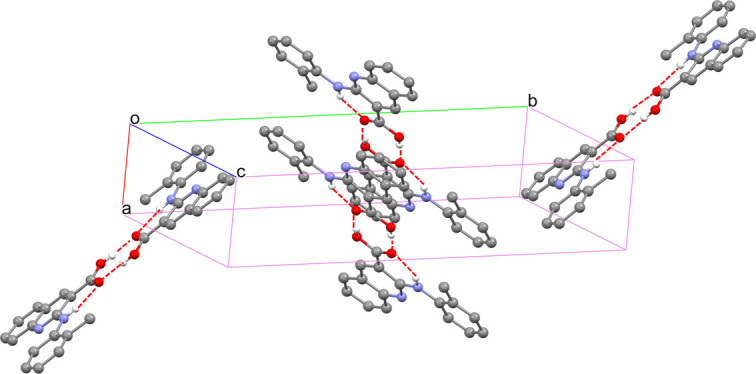
Packing of the mol­ecules in (**I**) with hydrogen bonds shown as red dashed lines (for clarity, H atoms not involved in hydrogen bonding are omitted).

Compound (**I**) (Fig. 1 ▸) is an analogue of 2-(phenyl­amino)­nicotinic acid (Long *et al.*, 2008 ▸), which is a compound with rich polymorphism. In the new compound, a quinoline ring replaces the pyridine ring. The C1–C9/N1 quinoline and pendant C10–C15 phenyl rings are almost coplanar, with a dihedral angle of 6.19 (6)° and an intra­molecular N—H⋯O hydrogen bond (Table 1 ▸) supports the near-planar conformation. In the extended structure, the mol­ecules associate to form centrosymmetric carb­oxy­lic-acid dimers linked by pairs of O—H⋯O hydrogen bonds (Fig. 2 ▸).

## Synthesis and crystallization

**Figure 3:**
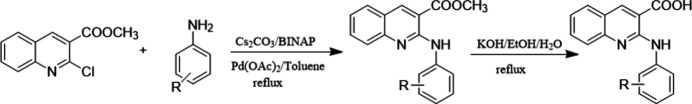
Synthesis scheme for (**I**).

The title compound was synthesized in two steps using a Buchwald–Hartwig cross-coupling reaction and a hydrolysis reaction (Fig. 3 ▸). The compound was purified by column chromatography. Single crystals in the form of yellow needles were obtained by slowly evaporating an acetone solution of the title compound.

## Refinement

Crystal data, data collection and structure refinement details are summarized in Table 2 ▸.

## Supplementary Material

Crystal structure: contains datablock(s) global, I. DOI: 10.1107/S2414314626005547/hb4562sup1.cif

Structure factors: contains datablock(s) I. DOI: 10.1107/S2414314626005547/hb4562Isup3.hkl

CCDC reference: 2556750

Additional supporting information:  crystallographic information; 3D view; checkCIF report

## Figures and Tables

**Table 1:** Hydrogen-bond geometry (Å, °)

*D*—H⋯*A*	*D*—H	H⋯*A*	*D*⋯*A*	*D*—H⋯*A*
N2—H2⋯O2	0.86	1.97	2.6956 (14)	141
O1—H1⋯O2^i^	0.82	1.85	2.6684 (13)	177

Symmetry code: (i) 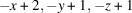.

**Table 2:** Experimental details

Crystal data	
Chemical formula	C_17_H_14_N_2_O_2_
*M*_r_	278.30
Crystal system, space group	Monoclinic, *P*2_1_/*n*
Temperature (K)	300
*a*, *b*, *c* (Å)	4.9122 (1), 24.0804 (6), 11.5773 (2)
β (°)	90.220 (2)
*V* (Å^3^)	1369.44 (5)
*Z*	4
Radiation type	Cu *K*α
μ (mm^−1^)	0.73
Crystal size (mm)	0.2 × 0.08 × 0.07

Data collection	
Diffractometer	ROD, Synergy Custom system, HyPix
Absorption correction	Multi-scan (*CrysAlis PRO*; Rigaku OD, 2024 ▸)
*T*_min_, *T*_max_	0.606, 1.000
No. of measured, independent and observed [*I* > 2σ(*I*)] reflections	11254, 2830, 2380
*R*_int_	0.032
(sin θ/λ)_max_ (Å^−1^)	0.633

Refinement	
*R*[*F*^2^ > 2σ(*F*^2^)], *wR*(*F*^2^), *S*	0.040, 0.121, 1.06
No. of reflections	2830
No. of parameters	193
H-atom treatment	H-atom parameters constrained
Δρ_max_, Δρ_min_ (e Å^−3^)	0.19, −0.14

Computer programs: *CrysAlis PRO* (Rigaku OD, 2024 ▸), *SHELXT* (Sheldrick, 2015*a* ▸), *SHELXL2018/3* (Sheldrick, 2015*b* ▸) a *OLEX2* (Dolomanov *et al.*, 2009 ▸).

## full crystallographic data

### 2-[(2-Methylphenyl)amino]quinoline-3-carboxylic acid Crystal data

**Table:**

C_17_H_14_N_2_O_2_	*F*(000) = 584
*M**_r_* = 278.30	*D*_x_ = 1.350 Mg m^−^^3^
Monoclinic, *P*2_1_/*n*	Cu *K*α radiation, λ = 1.54184 Å
*a* = 4.9122 (1) Å	Cell parameters from 6020 reflections
*b* = 24.0804 (6) Å	θ = 3.7–77.1°
*c* = 11.5773 (2) Å	µ = 0.73 mm^−^^1^
β = 90.220 (2)°	*T* = 300 K
*V* = 1369.44 (5) Å^3^	Needle, clear dark yellow
*Z* = 4	0.2 × 0.08 × 0.07 mm

### 2-[(2-Methylphenyl)amino]quinoline-3-carboxylic acid Data collection

**Table:**

ROD, Synergy Custom system, HyPix diffractometer	2830 independent reflections
Radiation source: Rotating-anode X-ray tube, Rigaku (Cu) X-ray Source	2380 reflections with *I* > 2σ(*I*)
Mirror monochromator	*R*_int_ = 0.032
Detector resolution: 10.0000 pixels mm^-1^	θ_max_ = 77.6°, θ_min_ = 3.7°
ω scans	*h* = −5→6
Absorption correction: multi-scan (CrysAlisPro; Rigaku OD, 2024)	*k* = −30→30
*T*_min_ = 0.606, *T*_max_ = 1.000	*l* = −14→14
11254 measured reflections	

### 2-[(2-Methylphenyl)amino]quinoline-3-carboxylic acid Refinement

**Table:**

Refinement on *F*^2^	Hydrogen site location: inferred from neighbouring sites
Least-squares matrix: full	H-atom parameters constrained
*R*[*F*^2^ > 2σ(*F*^2^)] = 0.040	*w* = 1/[σ^2^(*F*_o_^2^) + (0.0605*P*)^2^ + 0.1663*P*] where *P* = (*F*_o_^2^ + 2*F*_c_^2^)/3
*wR*(*F*^2^) = 0.121	(Δ/σ)_max_ < 0.001
*S* = 1.06	Δρ_max_ = 0.19 e Å^−^^3^
2830 reflections	Δρ_min_ = −0.14 e Å^−^^3^
193 parameters	Extinction correction: SHELXL2018/3 (Sheldrick 2015b), Fc^*^=kFc[1+0.001xFc^2^λ^3^/sin(2θ)]^-1/4^
0 restraints	Extinction coefficient: 0.0043 (8)
Primary atom site location: dual	

### 2-[(2-Methylphenyl)amino]quinoline-3-carboxylic acid Special details

**Table:**

Geometry. All esds (except the esd in the dihedral angle between two l.s. planes) are estimated using the full covariance matrix. The cell esds are taken into account individually in the estimation of esds in distances, angles and torsion angles; correlations between esds in cell parameters are only used when they are defined by crystal symmetry. An approximate (isotropic) treatment of cell esds is used for estimating esds involving l.s. planes.
Refinement. The H atoms were placed in idealized locations and refined as riding atoms.

### 2-[(2-Methylphenyl)amino]quinoline-3-carboxylic acid Fractional atomic coordinates and isotropic or equivalent isotropic displacement parameters (Å^2^)

**Table:**

	*x*	*y*	*z*	*U*_iso_*/*U*_eq_	
O1	0.86071 (19)	0.50007 (4)	0.64825 (8)	0.0544 (3)	
H1	0.972645	0.514724	0.605628	0.082*	
O2	0.76184 (18)	0.45416 (4)	0.48683 (8)	0.0528 (3)	
N1	0.1504 (2)	0.36553 (5)	0.67276 (10)	0.0489 (3)	
N2	0.3664 (2)	0.37672 (5)	0.49696 (10)	0.0514 (3)	
H2	0.493564	0.394442	0.461808	0.062*	
C1	0.3361 (2)	0.39115 (5)	0.60958 (11)	0.0434 (3)	
C2	0.5062 (2)	0.43478 (5)	0.65729 (10)	0.0427 (3)	
C3	0.4650 (3)	0.44993 (6)	0.76930 (11)	0.0487 (3)	
H3	0.571032	0.478019	0.801326	0.058*	
C4	0.2651 (3)	0.42393 (6)	0.83770 (11)	0.0485 (3)	
C5	0.2115 (3)	0.43787 (8)	0.95348 (13)	0.0656 (4)	
H5	0.306527	0.466831	0.988248	0.079*	
C6	0.0207 (3)	0.40921 (8)	1.01528 (13)	0.0688 (4)	
H6	−0.013247	0.418603	1.091801	0.083*	
C7	−0.1219 (3)	0.36607 (7)	0.96347 (14)	0.0636 (4)	
H7	−0.249664	0.346493	1.006340	0.076*	
C8	−0.0788 (3)	0.35180 (6)	0.85132 (13)	0.0569 (4)	
H8	−0.177882	0.322935	0.818292	0.068*	
C9	0.1164 (2)	0.38075 (5)	0.78466 (11)	0.0461 (3)	
C10	0.2272 (3)	0.33793 (6)	0.42729 (12)	0.0493 (3)	
C11	0.2962 (3)	0.33752 (6)	0.31000 (12)	0.0520 (3)	
C12	0.1663 (3)	0.29969 (7)	0.23774 (14)	0.0630 (4)	
H12	0.212688	0.298684	0.159966	0.076*	
C13	−0.0303 (4)	0.26344 (7)	0.27837 (17)	0.0706 (5)	
H13	−0.116345	0.238710	0.228478	0.085*	
C14	−0.0961 (4)	0.26463 (7)	0.39330 (17)	0.0692 (4)	
H14	−0.228135	0.240482	0.421178	0.083*	
C15	0.0303 (3)	0.30104 (7)	0.46839 (14)	0.0619 (4)	
H15	−0.015566	0.301025	0.546256	0.074*	
C16	0.5042 (3)	0.37707 (7)	0.26173 (13)	0.0626 (4)	
H16A	0.450583	0.414562	0.278322	0.094*	
H16B	0.516342	0.372110	0.179635	0.094*	
H16C	0.678339	0.369766	0.296465	0.094*	
C17	0.7195 (2)	0.46328 (5)	0.58959 (11)	0.0432 (3)	

### 2-[(2-Methylphenyl)amino]quinoline-3-carboxylic acid Atomic displacement parameters (Å^2^)

**Table:**

	*U*^11^	*U*^22^	*U*^33^	*U*^12^	*U*^13^	*U*^23^
O1	0.0490 (5)	0.0645 (6)	0.0498 (5)	−0.0156 (4)	0.0073 (4)	−0.0006 (4)
O2	0.0487 (5)	0.0648 (6)	0.0449 (5)	−0.0131 (4)	0.0084 (4)	0.0016 (4)
N1	0.0468 (6)	0.0531 (6)	0.0468 (6)	−0.0057 (5)	0.0081 (4)	0.0046 (5)
N2	0.0487 (6)	0.0606 (7)	0.0450 (6)	−0.0125 (5)	0.0084 (5)	−0.0009 (5)
C1	0.0383 (6)	0.0480 (6)	0.0439 (6)	0.0002 (5)	0.0035 (5)	0.0063 (5)
C2	0.0363 (6)	0.0493 (7)	0.0426 (6)	0.0002 (5)	0.0024 (5)	0.0065 (5)
C3	0.0444 (6)	0.0566 (7)	0.0450 (7)	−0.0059 (5)	0.0017 (5)	0.0022 (6)
C4	0.0438 (6)	0.0587 (8)	0.0430 (6)	0.0003 (5)	0.0047 (5)	0.0062 (5)
C5	0.0653 (9)	0.0845 (11)	0.0470 (8)	−0.0111 (8)	0.0093 (6)	−0.0048 (7)
C6	0.0693 (9)	0.0914 (12)	0.0458 (8)	−0.0020 (9)	0.0164 (7)	0.0040 (8)
C7	0.0584 (8)	0.0751 (10)	0.0576 (8)	−0.0008 (7)	0.0194 (7)	0.0149 (8)
C8	0.0542 (7)	0.0584 (8)	0.0584 (8)	−0.0066 (6)	0.0134 (6)	0.0084 (6)
C9	0.0420 (6)	0.0505 (7)	0.0460 (6)	0.0039 (5)	0.0063 (5)	0.0079 (5)
C10	0.0457 (6)	0.0515 (7)	0.0507 (7)	0.0004 (5)	0.0007 (5)	−0.0024 (6)
C11	0.0498 (7)	0.0555 (7)	0.0506 (7)	0.0092 (6)	0.0005 (5)	−0.0038 (6)
C12	0.0714 (9)	0.0627 (9)	0.0550 (8)	0.0123 (7)	−0.0063 (7)	−0.0105 (7)
C13	0.0747 (10)	0.0566 (9)	0.0803 (12)	0.0010 (7)	−0.0198 (9)	−0.0141 (8)
C14	0.0666 (9)	0.0608 (9)	0.0802 (11)	−0.0133 (7)	−0.0060 (8)	−0.0029 (8)
C15	0.0618 (8)	0.0632 (9)	0.0606 (9)	−0.0130 (7)	0.0022 (7)	−0.0012 (7)
C16	0.0662 (9)	0.0747 (10)	0.0470 (7)	0.0003 (7)	0.0132 (6)	−0.0026 (7)
C17	0.0371 (6)	0.0488 (7)	0.0437 (6)	−0.0009 (5)	0.0000 (5)	0.0053 (5)

### 2-[(2-Methylphenyl)amino]quinoline-3-carboxylic acid Geometric parameters (Å, º)

**Table:**

O1—H1	0.8200	C7—H7	0.9300
O1—C17	1.3129 (16)	C7—C8	1.360 (2)
O2—C17	1.2284 (15)	C8—H8	0.9300
N1—C1	1.3239 (15)	C8—C9	1.4164 (17)
N1—C9	1.3573 (17)	C10—C11	1.4008 (19)
N2—H2	0.8600	C10—C15	1.398 (2)
N2—C1	1.3582 (17)	C11—C12	1.390 (2)
N2—C10	1.4096 (18)	C11—C16	1.506 (2)
C1—C2	1.4503 (18)	C12—H12	0.9300
C2—C3	1.3629 (18)	C12—C13	1.385 (3)
C2—C17	1.4797 (16)	C13—H13	0.9300
C3—H3	0.9300	C13—C14	1.371 (3)
C3—C4	1.4105 (18)	C14—H14	0.9300
C4—C5	1.408 (2)	C14—C15	1.380 (2)
C4—C9	1.410 (2)	C15—H15	0.9300
C5—H5	0.9300	C16—H16A	0.9600
C5—C6	1.368 (2)	C16—H16B	0.9600
C6—H6	0.9300	C16—H16C	0.9600
C6—C7	1.388 (3)		
			
C17—O1—H1	109.5	N1—C9—C8	118.25 (13)
C1—N1—C9	119.29 (12)	C4—C9—C8	118.43 (12)
C1—N2—H2	114.2	C11—C10—N2	116.15 (12)
C1—N2—C10	131.61 (11)	C15—C10—N2	124.14 (13)
C10—N2—H2	114.2	C15—C10—C11	119.72 (13)
N1—C1—N2	119.31 (12)	C10—C11—C16	121.48 (13)
N1—C1—C2	121.62 (12)	C12—C11—C10	118.41 (14)
N2—C1—C2	119.07 (11)	C12—C11—C16	120.11 (13)
C1—C2—C17	122.84 (11)	C11—C12—H12	119.1
C3—C2—C1	117.96 (11)	C13—C12—C11	121.83 (15)
C3—C2—C17	119.20 (12)	C13—C12—H12	119.1
C2—C3—H3	119.3	C12—C13—H13	120.5
C2—C3—C4	121.44 (13)	C14—C13—C12	118.91 (15)
C4—C3—H3	119.3	C14—C13—H13	120.5
C5—C4—C3	124.19 (14)	C13—C14—H14	119.4
C5—C4—C9	119.48 (12)	C13—C14—C15	121.18 (16)
C9—C4—C3	116.33 (12)	C15—C14—H14	119.4
C4—C5—H5	119.7	C10—C15—H15	120.0
C6—C5—C4	120.58 (16)	C14—C15—C10	119.94 (15)
C6—C5—H5	119.7	C14—C15—H15	120.0
C5—C6—H6	120.1	C11—C16—H16A	109.5
C5—C6—C7	119.81 (15)	C11—C16—H16B	109.5
C7—C6—H6	120.1	C11—C16—H16C	109.5
C6—C7—H7	119.3	H16A—C16—H16B	109.5
C8—C7—C6	121.42 (14)	H16A—C16—H16C	109.5
C8—C7—H7	119.3	H16B—C16—H16C	109.5
C7—C8—H8	119.9	O1—C17—C2	114.38 (11)
C7—C8—C9	120.26 (15)	O2—C17—O1	122.04 (11)
C9—C8—H8	119.9	O2—C17—C2	123.57 (11)
N1—C9—C4	123.31 (11)		
			
N1—C1—C2—C3	−1.65 (19)	C4—C5—C6—C7	−0.2 (3)
N1—C1—C2—C17	178.61 (11)	C5—C4—C9—N1	178.35 (13)
N2—C1—C2—C3	177.62 (12)	C5—C4—C9—C8	−1.5 (2)
N2—C1—C2—C17	−2.12 (18)	C5—C6—C7—C8	−0.8 (3)
N2—C10—C11—C12	179.45 (13)	C6—C7—C8—C9	0.5 (2)
N2—C10—C11—C16	−1.0 (2)	C7—C8—C9—N1	−179.25 (14)
N2—C10—C15—C14	179.76 (15)	C7—C8—C9—C4	0.6 (2)
C1—N1—C9—C4	1.7 (2)	C9—N1—C1—N2	−178.68 (12)
C1—N1—C9—C8	−178.45 (12)	C9—N1—C1—C2	0.59 (19)
C1—N2—C10—C11	174.13 (13)	C9—C4—C5—C6	1.3 (2)
C1—N2—C10—C15	−6.0 (2)	C10—N2—C1—N1	1.6 (2)
C1—C2—C3—C4	0.49 (19)	C10—N2—C1—C2	−177.73 (13)
C1—C2—C17—O1	−177.35 (11)	C10—C11—C12—C13	1.0 (2)
C1—C2—C17—O2	3.29 (19)	C11—C10—C15—C14	−0.3 (2)
C2—C3—C4—C5	−179.58 (14)	C11—C12—C13—C14	−0.7 (2)
C2—C3—C4—C9	1.5 (2)	C12—C13—C14—C15	−0.1 (3)
C3—C2—C17—O1	2.91 (17)	C13—C14—C15—C10	0.6 (3)
C3—C2—C17—O2	−176.45 (12)	C15—C10—C11—C12	−0.5 (2)
C3—C4—C5—C6	−177.53 (15)	C15—C10—C11—C16	179.12 (14)
C3—C4—C9—N1	−2.7 (2)	C16—C11—C12—C13	−178.60 (15)
C3—C4—C9—C8	177.40 (12)	C17—C2—C3—C4	−179.76 (12)

### 2-[(2-Methylphenyl)amino]quinoline-3-carboxylic acid Hydrogen-bond geometry (Å, º)

**Table:**

*D*—H···*A*	*D*—H	H···*A*	*D*···*A*	*D*—H···*A*
N2—H2···O2	0.86	1.97	2.6956 (14)	141
O1—H1···O2^i^	0.82	1.85	2.6684 (13)	177

Symmetry code: (i) −*x*+2, −*y*+1, −*z*+1.
